# Isotretinoin use in rhinoplasty patients

**DOI:** 10.1016/j.bjorl.2025.101592

**Published:** 2025-03-26

**Authors:** Jholbert Cardoso Santana, Hugo Valter Ramos Lisboa, Leandro Azevedo de Camargo, Ricardo Araújo Meira Almeida, Lourival Mendes Bueno

**Affiliations:** Hospital das Clínicas da Universidade Federal de Goiás (HC-UFG), Departamento de Otorrinolaringologia e Cirurgia de Cabeça e Pescoço, Goiânia, GO, Brazil

**Keywords:** Isotretinoin, Rhinoplasty, Thick skin

## Abstract

**Objective:**

Presenting evidence available on perioperative isotretinoin application in, and benefits for, thick-skinned patients subjected to rhinoplasty.

**Methods:**

Analysis was applied to articles available in the following databases: Cochrane, PubMed, ScienceDirect and Scielo. The analyzed corpus comprised studies published from 2004 to 2023 focused on investigating thick-skinned patients subjected to rhinoplasty and treated with isotretinoin. Inappropriate and unrelated methodologies were excluded from the analysis.

**Results:**

Initially, 1,214 articles were collected, but this number dropped to 617 after duplicates’ removal. Twenty (20) articles remained in the corpus after inclusion criteria application, but 10 of them were excluded from the analysis based on exclusion criteria. Finally, 10 articles were selected for review purposes: 1 experience report, 2 case studies, 3 narrative reviews and 4 clinical trials.

**Conclusions:**

The current findings suggested aesthetic improvements in patients treated with isotretinoin, although its long-term benefits remain uncertain. Thus, further research on this topic should be conducted.

## Highlights

• It is possible concluding that isotretinoin has potential aesthetic benefits.
• Studies have pointed towards improved skin appearance and nasal tip definition.
• However, these benefits may be more visible in the initial postoperative period.
• There are no protocols in the literature regarding dosage and duration of treatment.

## Introduction

Rhinoplasty presents significant challenges. Nasal anatomy complexity, individual variations in healing and patients’ expectations contribute to difficulties associated with this procedure, whose potential complications comprise, but are not limited to, bleeding, infection, irregularities in nose shape, breathing issues and dissatisfaction with cosmetic appearance. Suboptimal healing can lead to unwanted aesthetic outcomes, such as visible scars, asymmetries and irregular contours.^1^, ^2^

The skin features in the perioperative period of rhinoplasty patients play key role in healing process and aesthetic success. Individuals with oily or acne-prone skin may experience remarkably complex recovery process due to higher risk of presenting clogged pores, cysts and inflammation. In addition, thick-skinned patients may find it hard to get accurate nasal contour definition. Improvements in skin texture and quality can potentially enhance rhinoplasty-related aesthetic outcomes by promoting smoother and even skin surface.^3^

Isotretinoin has been used to improve skin quality, since it has the potential to enhance the quality of post-rhinoplasty wounds. This drug, which is a vitamin-A derivative, was introduced in the pharmaceutical market in the 1980s and it has revolutionized the treatment applied to severe acne, mainly to that resistant to other therapeutic modalities. This systemic retinoid is acknowledged for its remarkable effectiveness in controlling acne, suppressing bacterial proliferation and in mitigating inflammation. The isotretinoin action mechanism is based on normalizing keratinocytes’ differentiation to reduce the formation of comedones. In addition, isotretinoin has antiproliferative and pro-apoptotic effect on sebaceous gland cells, besides significantly decreasing sebum production.^4^

Furthermore, studies have shown that isotretinoin can positively influence collagen remodeling, mainly in thick skin. Collagen is key component in wound healing and in maintaining skin structural integrity. Isotretinoin boosts the healing process and improves nasal tip definition by optimizing collagen remodeling. This aspect is mostly relevant in rhinoplasty procedures, since scars and definition points play key role in aesthetic outcomes.^5^

However, isotretinoin can adversely affect wound healing, which is a critical factor for the postoperative period of patients subjected to rhinoplasty. It is so, because isotretinoin decreases sebum production and changes the function of sebaceous glands. Consequently, patients’ skin gets drier and less elastic, and it increases the risk of postoperative complications, such as hypertrophic scars or keloids. Furthermore, isotretinoin can interfere with both angiogenesis and patients’ inflammatory response, which are factors playing essential role in effective healing processes. Therefore, plastic surgeons must take into consideration the likelihood of adverse effects deriving from isotretinoin application in rhinoplasty patients and, therefore, closely monitor these patients’ postoperative healing process.^4^

The isotretinoin side-effect profile, which comprises xeroderma, cheilitis, potential hepatotoxicity and teratogenicity, requires close clinical and laboratory monitoring. It is essential monitoring patients’ serum lipids and liver enzymes, given the association between isotretinoin-induced hyperlipidemia and risk of developing pancreatitis. Furthermore, strict contraception methods are mandatory in women undergoing isotretinoin-based treatment.^6^

Since the 1980s, most studies in the literature have advocated against skin surgery procedures associated with isotretinoin application. It was so because, assumingly, this drug could have negative effect on surgical wound healing; therefore, scholars recommended a 12-mo interval (at least) between isotretinoin-use suspension and surgery. A meta-analysis was published in 2017 after reviewing the literature published on this topic. None of the 1,485 analyzed studies presented sufficient and convincing evidence to support the recommendation to postpone surgical procedures in patients undergoing concomitant isotretinoin therapy or in those subjected to it 6 to 12 mo before surgery.^7^

The current scientific literature presents several gaps when it comes to the actual benefits and likely risks associated with using retinoids in thick-skinned patients subjected to rhinoplasty. Thus, the aim of the current study was to perform a scoping review to gather current knowledge about this topic. It was done to present evidence available on the application and benefits of retinoids in this specific context. Although there are literature reviews on this topic, clinical trials that have not yet been approached and addressed have emerged.

## Methods

### Study type

Scoping review.

### Review questions

Is there any benefit associated with isotretinoin application to treat thick skin in the perioperative period of patients subjected to rhinoplasty?

How should isotretinoin be used to treat thick skin in the perioperative period of patients subjected to rhinoplasty?

### Defining inclusion and exclusion criteria

Inclusion: Studies addressing isotretinoin application in association with rhinoplasty. Studies published in the last 20 years, i.e., from January 2004 to December 2023.

Exclusion: Studies conducted with non-human individuals, studies presenting inadequate methodology, opinion articles lacking empirical data and publications written in languages other than English, Portuguese or Spanish.

### Search strategy

Electronic databases, such as Cochrane, PubMed, ScienceDirect and Scielo, were herein used.

Keywords included combinations of terms, such as “isotretinoin”, “rhinoplasty” and “thick skin”.

The PubMed platform was initially used to define the mesh terms, which were then used to perform a standard search in the other databases.

### Selection process

Initially, the titles and abstracts of articles found in the searched databases were reviewed to check their compliance with the inclusion criteria.

Articles selected at this stage were fully read for in-depth assessment purposes.

### Data extraction

Data, such as publication year, study design, sample size, participants’ features, isotretinoin treatment details, rhinoplasty outcomes and main conclusions, were extracted from the analyzed studies.

### Data synthesis

Data were synthesized in a narrative way to highlight the main discoveries, trends and gaps in the literature.

### Discussion and conclusion

Results were discussed based on existing evidence by taking into consideration clinical implications and recommendations for future practice.

Study limitations and fields for future research were also addressed.

## Results

**Fig. 1:**
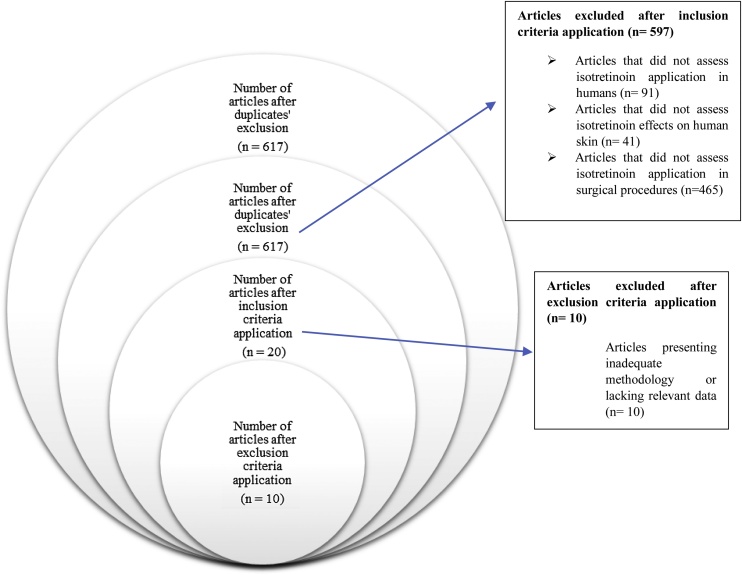
Data analysis flowchart.

In total, 1,214 articles on the investigated topic, published between January 2004 and December 2023 (Table 1), were collected: 1,125 of them were found in the Cochrane database, whereas the other 89 articles were collected in the other databases. After duplicated articles were excluded, 617 publications remained in the corpus. Inclusion criteria application reduced the number of articles by 597. Ten (10) of the remaining 20 articles were excluded after exclusion criteria application. Finally, 10 articles were subjected to the current review (Fig. 1).

**Table 1:** Number of articles based on each search algorithm in the Cochrane, PubMed, ScienceDirect and Scielo databases.

Search terms	Number of articles found in each database				Total
	Cochrane	PubMed	ScienceDirect	Scielo	
Isotretinoin OR 13 cis Retinoic Acid OR Roaccutane OR Accutane AND Rhinoplasty	563	13	43	1	620
Isotretinoin OR 13 cis Retinoic Acid OR Roaccutane OR Accutane) AND Rhinoplasty AND Thick skin	562	9	22	1	594
Total	1.125	22	65	2	1.214

**Fig. 2:**
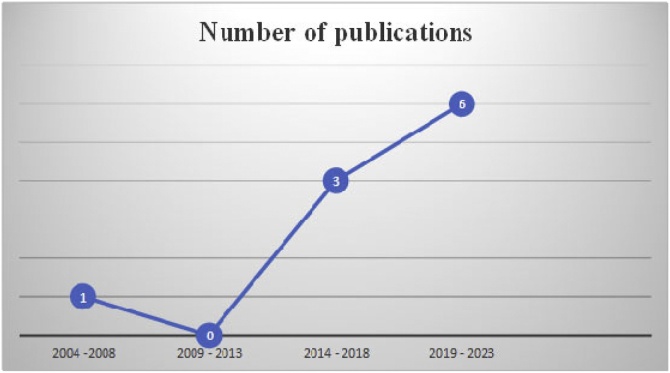
Number of selected articles based on publication year.

One (1) of all 10 analyzed articles was an experience report, 2 were case studies, 3 were narrative literature reviews and 4 were clinical trials (Fig. 2 and Table 2).

**Table 2:** Number of articles published per country.

Country	Number of articles
Turkey	1
Brazil	1
Colombia	3
United States of America	2
Iran	3

## Discussion

The current literature about isotretinoin application in patients subjected to rhinoplasty is both vast and diverse. It addresses a wide range of aspects that go from potential benefits to specific concerns about patient’s healing and aesthetic outcomes.

A case study published in 2005 investigated three patients who started using isotretinoin after rhinoplasty. This publication raised the hypothesis of causal correlation between this medication and complications during surgical procedure. This study pointed towards postoperative nasal-tip deformities requiring additional surgical interventions.^8^ At that time, this research cast significant doubt on the benefits of using isotretinoin to treat thick skin in rhinoplasty patients. Researchers in this publication suggested the likely association between using this medication and future complications. However, subsequent in-depth investigations questioned this causal connection by arguing that the observed complications could have emerged from the adoption of inadequate surgical techniques rather than from isotretinoin using.^9^

In 2016, a Colombian researcher published a pioneer case study addressing isotretinoin using over rhinoplasty perioperative period. She assessed 17 patients (7 men and 10 women) with thick acne-prone skin who were treated with oral isotretinoin doses ranging from 0.25 to 0.5 mg/kg, for 4 to 6 mo, after surgery. These patients were followed-up through periodic photos taken after surgery. They presented improved skin appearance and texture, nasal tip definition and sebum production control, as well as no reports of abnormal scars.^10^ The aforementioned researcher has subjectively performed these assessments, since her study did not report the application of satisfaction questionnaires or objective assessments.

The aforementioned researcher published another article in 2018, which corroborated the current study. Her study was an experience report focused on assessing and classifying patients’ skin based on its thickness. Furthermore, she established a treatment regimen based on low isotretinoin doses (from 0.25 to 0.4 mg/kg/day) to optimize aesthetic outcomes in thick-skinned patients. Treatment started 4 to 6 weeks before surgery and stopped 1 week before surgery. Isotretinoin application restarted 2 to 3 weeks after surgery and went on for approximately 4 mo. Significant improvements in aesthetic outcomes were observed, mainly in patients with oily and acne-prone skin.^11^

Recently, some experimental studies have added to the literature by addressing the real benefit of isotretinoin application in thick-skinned patients subjected to rhinoplasty. A double-blind clinical trial comprising 48 cases, published in 2018, assessed oral isotretinoin effectiveness in treating postoperative edema. The treatment started 31 days after surgery, based on 0.5 mg/kg isotretinoin administration every other day in the first month of treatment and then, on its daily application for additional 2 mo. Assessments were based on questionnaires filled out by patients and on analyses (performed by the surgeon) of serial photos taken before and after surgery. Improved patient satisfaction and aesthetic outcomes were observed in the first 3 and 6 postoperative months, although these parameters did not show significant difference after 12-mo.^12^ This finding suggests that isotretinoin can help improving initial aesthetic outcomes in thick-skinned patients subjected to rhinoplasty, but it does not affect these outcomes one year after surgery.

Another analytical study, published in 2020, involved 303 patients subjected to rhinoplasty between 2012 and 2015. It assessed isotretinoin impact on patients’ perioperative period. Patients were divided into control and experimental groups. The experimental group was treated with isotretinoin (20 mg/day) from two weeks before to two months after surgery. Questionnaires were applied in the 1^st^, 3^rd^, 6^th^ and 12^th^ months after surgery to assess patients’ satisfaction. In addition, both the surgeon and the dermatologist carried out objective assessments within the same period. The authors of the aforementioned study highlighted the effectiveness of this medication in reducing skin thickness, oiliness and acne in the 3^rd^ and 6^th^ postoperative months. However, there was neither statistical difference in these improvements 1 year after surgery, nor impairment in skin healing or cartilaginous deformities.^7^ This study was in compliance with the previous one, since it suggested that isotretinoin may be safe and help improving patients’ satisfaction in the first months after rhinoplasty surgery.

Another randomized double-blind study published in 2023 investigated oral isotretinoin administration effectiveness as adjuvant treatment in rhinoplasty cases. In total, 24 patients included in the study were divided into control and intervention groups. The intervention group was treated with isotretinoin (20 mg/day ‒ equivalent to 0.25–0.4 mg/kg/day) for 2 mo before surgery, stopped taking it 15 days before rhinoplasty surgery and resumed medication 3 to 5 days after the procedure for additional 4 mo. High-frequency ultrasound assessment has evidenced significant reduction in epidermis and dermis thickness in the intervention group, mainly in the nasal dorsum and nasal ala, 6 mo after rhinoplasty. A satisfaction questionnaire was also applied to patients. Both assessments were carried out before patients started pre-operatively taking this medication and 6 mo after surgery. Isotretinoin was effective in reducing skin thickness and recorded the highest patient satisfaction scores. This outcome suggested that this medication is a beneficial treatment option to be used in association with rhinoplasty. No healing issues or deformities were observed in the intervention group.^13^ Although this study has evidenced isotretinoin’s beneficial action, the analysis was only carried out 6 mo after surgery, and it did not enable establishing isotretinoin effect on patients’ late outcomes.

Furthermore, a prospective study (published in 2021) conducted with 40 patients focused on analyzing isotretinoin effect on nasal skin thickness and elasticity in patients with acne vulgaris, who were treated with oral isotretinoin doses (0.25 mg/kg/day, n = 16 or 0.5 mg/kg/day, n = 24) for 4 mo and subjected to serial ultrasound and elastography assessments. Significant reduction in dermis and nasal subcutaneous tissue thickness, as well as increased skin elasticity, were observed. Therefore, the aforementioned study suggested using isotretinoin as effective treatment to help improving nasal skin quality in rhinoplasty patients, although there was no statistically significant difference between the adopted isotretinoin doses.^14^

Three literature reviews on the isotretinoin-rhinoplasty topic were recently published. The first article highlighted the relevance of dermatological preparation and interdisciplinary collaboration between plastic surgeons and dermatologists, as well as proposed an off-label isotretinoin regimen to help optimizing aesthetic outcomes in thick and sebaceous skin cases.^15^ This approach was completed by findings in the second article, which also highlighted isotretinoin potential benefits in improving skin texture and nasal definition, although it acknowledged the risks associated with isotretinoin application, such as healing issues and scar formation.^16^ Finally, the third study addressed the controversy around isotretinoin using in rhinoplasty cases. This study acknowledged this medication’s effectiveness in reducing skin thickness and in improving its elasticity, but it highlighted the importance of adopting a personalized approach by taking into account isotretinoin’s side effects and patients’ medical history.^17^

In this context, the review of review articles proved to be highly beneficial for the development of this academic work, even though these articles do not present primary data from an original study group. By extensively synthesizing existing literature, these reviews provided a comprehensive overview of the current state of knowledge regarding the potential effects of isotretinoin on patients undergoing rhinoplasty. This approach allowed for the identification of significant gaps in the literature, a critical assessment of different methodological and conceptual perspectives on the subject, and the formulation of better-grounded recommendations for pre- and postoperative management of these patients. Thus, the analysis of review articles not only facilitated the construction of a solid theoretical foundation on the potential benefits of isotretinoin in the surgical context but also contributed to the development of a more robust and coherent methodological approach throughout the work.

Furthermore, according to the literature, concomitant use of medications must be thoroughly assessed before starting isotretinoin administration. Tetracyclines are contraindicated in these cases due to risk of pseudotumor cerebri development. Vitamin A supplements can be toxic in interactions with carbamazepine, ketoconazole, salicylic acid and indomethacin, as reported in the literature. Alcohol intake can decrease isotretinoin effectiveness, whose well-known teratogenic potential requires adopting strict contraception methods. Women should abstain from sexual activity or use two contraception methods, from 1 mo before to 1 mo after isotretinoin administration. In addition, serum lipids and liver function should be monitored. Contraindications to this medication comprise hypersensitivity to isotretinoin, to its components and to vitamin A, pregnancy, lactation and being under 12 years old.^6^ It is essential clinically monitoring side effects during rhinoplasty perioperative period, when homeostasis and skin quality play key role in aesthetic outcomes.

Finally, the current literature is limited by several factors, such as sample size, diversity of patient groups and variability in research methodologies. Many studies are retrospective or based on case reports, and it can lead to biases and limit result extrapolation processes. There is clear need of conducting further prospective, randomized, controlled studies to enable a more robust and detailed understanding of isotretinoin effects on rhinoplasty cases.

Briefly, these studies provided a comprehensive overview on isotretinoin administration in rhinoplasty cases, as well as highlighted its potential effectiveness in improving patients’ satisfaction and nasal aesthetics, mainly in thick skin cases. However, studies presenting greater evidence have suggested that these benefits can only be seen in the initial results and that their long-term benefits remain questionable. Furthermore, there is no isotretinoin administration golden standard regarding treatment duration, dose and when to start taking it. Finally, individual management must be applied to patients taking isotretinoin, mainly with respect to its contraindications and side effects (Table 3).

**Table 3:** Comparison of studies about isotretinoin administration in thick-skinned patients subjected to rhinoplasty.

Publication year	Sample size	Isotretinoin dose	Treatment duration	Treatment description	Assessment	Final outcome
2005	3 patients	Not specified	6 mo	Isotretinoin used after surgery	Not specified	Hypothesis raised about causal correlation between isotretinoin and complications (nasal-tip deformities).
2016	17 patients	0.25–0.5 mg/kg	4 to 6 mo	Isotretinoin used after surgery	Follow-up through periodic photos, in the 6^th^ and 12^th^ month after surgery.	Improved skin appearance and no abnormal scars.
2018	Not specified	0.25–0.4 mg/kg/day	4 to 6 mo	Treatment started 4- to 6- weeks before surgery and stopped 1-week before surgery. Isotretinoin application restarted 2- to 3-weeks after surgery and went on for approximately 4-mo.	Classification of skin thickness and patient follow-up.	Significant improvements in aesthetic outcomes were observed, mainly in patients with oily and acne-prone skin.
2018	48 cases	0.5 mg/kg	3-mo	It started 31-days after surgery, on alternate days, in the first month and, then, on a daily basis	Patient satisfaction questionnaires and analysis of pre and postoperative photos by the surgeon	Improved patient satisfaction and aesthetic outcomes in the first 3 and 6 postoperative months, but no difference in these parameters after 12 mo
2020	303 patients	20 mg/day	2-mo and 2 weeks	It started 2-weeks before surgery and ended 2-mo after it, without interruption for surgery purposes	Patient satisfaction questionnaires were applied in the 1^st^, 3^rd^, 6^th^ and 12^th^ month after surgery. In addition, both the surgeon and the dermatologist carried out objective assessments in the same period.	Reduced skin thickness, oiliness and acne in the 3^rd^ and 6^th^ postoperative months; no significant differences in these parameters were observed 1 year after surgery.
2023	24 patients	20 mg/day (0.25–0.4 mg/kg/day)	6-mo	It started 2-mo before surgery, but it was interrupted 15 days before the procedure and resumed 3 to 5 days after surgery for additional 4-mo	High-frequency ultrasound assessment and patient satisfaction questionnaire were applied before patients started taking this medication and 6-mo after surgery.	Reduced epidermis and dermis thickness on both nasal bridge and ala, 6-mo after surgery; higher patient satisfaction; and no long-term effects.
2021	40 patients	0.25 mg/kg/day or 0.5 mg/kg/day	4-mo	It was applied for 4-mo in patients with acne vulgaris	Serial ultrasound and elastography assessments were performed before patients started taking this medication, as well as in 2^nd^ and 4^th^ months of treatment	Significantly reduced thickness in nasal dermis and subcutaneous tissue; increased skin elasticity, without significant difference between the adopted doses.

## Conclusion

According to the comprehensive literature review on isotretinoin administration in the perioperative period of patients subjected to rhinoplasty procedure, it is possible concluding that isotretinoin has potential aesthetic benefits, mainly in thick-skinned and acne-prone patients. Studies have pointed towards improved skin appearance and texture, nasal tip definition and sebum production control. However, their findings have pointed out that these benefits can be more visible in patients’ initial outcomes and that their long-term effects remain unclear. Moreover, lack of golden standard dose and treatment duration protocols highlights the need of adopting individualized and careful approaches. Secure isotretinoin management processes require closer attention to contraindications, side effects and drug interactions, with emphasis on the importance of performing rigorous assessment and clinical follow-up. Although promising, the current literature review highlights the need of conducting further controlled, randomized and prospective studies to set clear guidelines and to optimize isotretinoin administration in rhinoplasty cases to ensure patients’ satisfactory and safety outcomes.

## Funding

This research did not receive any specific grant from funding agencies in the public, commercial, or not-for-profit sectors.

## Declaration of competing interest

The authors declare no conflicts of interest.
